# Perspectives for a Framework to Understand Aril Initiation and Development

**DOI:** 10.3389/fpls.2016.01919

**Published:** 2016-12-20

**Authors:** Sylvia R. Silveira, Marcelo C. Dornelas, Adriana P. Martinelli

**Affiliations:** ^1^Laboratório de Biotecnologia Vegetal, Centro de Energia Nuclear na Agricultura, Universidade de São PauloPiracicaba, Brazil; ^2^Departamento de Biologia Vegetal, Instituto de Biologia, Universidade Estadual de CampinasCampinas, Brazil

**Keywords:** aril development, integument, model species, ovule, *Passiflora*, seed

## Abstract

A differentiated structure called “aril” has been described in seeds of several plant species during the course of evolution and might be considered as a supernumerary integument. Besides its ecological function in seed dispersal, the structure also represents a relevant character for systematic classification and exhibits important properties that impart agronomic value in certain species. Little is known about the molecular pathways underlying this morphological innovation because it is absent in currently used model species. A remarkable feature of the seeds of *Passiflora* species is the presence of a conspicuous aril. This genus is known for the ornamental, medicinal, and food values of its species. In view of the molecular resources and tools available for some *Passiflora* species, we highlight the potential of these species as models for developmental studies of the aril.

## Introduction

The morphological diversity among plant species results from differential gene expression controlling the development of novel features that ensure the adaptation and reproductive success of a species. An important question in plant biology is when and how these features emerged during evolution. One of such novel features is the aril. The aril is a differentiated structure present in seeds of several gymnosperm and angiosperm species, forming seed dispersal units. In many species, the aril accumulates several nutritional compounds attracting and rewarding frugivorous animals. There is a great amount of information available about morphological and molecular development of plant ovules and seeds and they can be used as initial clues to investigate aril development. These appendages are often used in systematics classification, since its presence, absence, form, and function vary among taxa. The well known model species do not exhibit this feature evidencing the need for novel models to study this specific structure. A better understanding of the processes involved in aril origin and development is interesting and necessary due to its economical, ecological and phylogenetic importance.

## Aril Origin and Importance

Several plant species develop differentiated structures associated with their seeds, often constituting diaspores, which are plant dispersal units mostly related to their dispersion syndrome (Corner, 1976). Some authors also believe that these structures originated as a protection mechanism for seeds and embryos, regardless of their role in dispersion (Mack, 2000). Also associated to the ovule/seed, either one or more integuments are found. The current theory of the evolution of integuments states that there are different evolutionary origins for the outer and inner integuments in flowering plants (Endress, 2011). In angiosperms, the inner integument is considered homologous to the single integument of extant and fossil gymnosperms (Reinheimer and Kellogg, 2009), and the outer integument may have been derived from a cupule/leaf-like structure found in several gymnosperms (Gasser et al., 1998). The integuments may or may not originate appendages that perform a defined role in seed dispersion. Such seed appendages may be wings, spines, hairs, plumes, fibers, or fleshy tissues, receiving different denominations in the literature.

Both gymnosperms and angiosperms evolved the habit of enveloping the seeds with a fleshy tissue (Lovisetto et al., 2012). Such tissue, called “aril,” generally accumulate sugars and other substances that will confer biological roles similar to those of fruits (Herrera, 1989).

**FIGURE 1:**
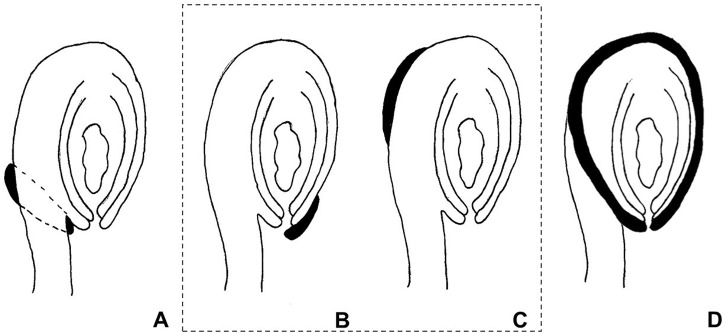
**Anatomical origin of different fleshy seed appendages or aril-like structures.** Areas in black represent the fleshy structures. **(A)** True aril – develops from the funiculus as a ring around it. **(B)** Caruncle – develops from the micropilar region of the integument. **(C)** Strophiole – develops from the raphe. **(D)** Sarcotesta – involves the differentiation of the outermost layer of the outer integument after fertilization. In the dashed box **(B,C)**, structures considered as “arillodes.”

The use of the term “aril” is quite controversial in the literature. It has been used both in a broader sense, referring to any fleshy structure associated with the seed, but also to designate structures with a specific anatomical origin. According to Corner (1976), the term defines a structure varying from a fleshy to a more-or-less hard consistency, which develops from part of the ovule after fertilization and envelopes the seed partially or completely. Van der Pijl (1972) preferred to distinguish these structures according to their anatomical origin, the aril being originated from the funiculus. Therefore, a structure developing from other parts of the ovule are usually called arillode, false aril, or aril-like structure (**Figure 1**). Both, aril and arillode, are somehow associated with integuments. In fact, some authors consider the “true” aril as a supernumerary integument (Maheshwari, 1950; Kapil and Vasil, 1963; Endress, 2011).

As arils are generally fleshy structures, they are of extreme importance because during their development and ripening they accumulate substances that confer properties that not only attract dispersion agents, but also arouse interest for human consumption. Arils are very common in tropical and subtropical species and might accumulate oils (e.g., *Ricinus communis*), flavor- and aroma-rich compounds (*Myristica fragrans*), nutrients, and sugars (*Passiflora edulis*), among other substances.

## Aril Ontogeny

Few studies describe the ontogeny and/or morphological aspects of aril formation and associate these with ovule development; this lack of information probably led to its controversial nomenclature. Additionally, the current model plant species do not exhibit this unique structure, making it difficult to characterize its development, especially at the molecular level.

Aril developmental stages were observed in some species of *Passiflora* (Raju, 1956; Singh, 1962; Dathan and Singh, 1973), and described in greater detail in *P. suberosa* and *Turnera ulmifolia* (Kloos and Bouman, 1980). It has also been described in Leguminosae, such as *Eriosema glaziovii* (Grear and Dengler, 1976), *Cytisus striatus*, and *C. multiflorus* (Rodriguez-Riaño et al., 2006). More recently, the development of an aril was described in Celastraceae, however, the authors showed that the origin of the aril-like structure was not from the funiculus, calling it “caruncula” (Zhang et al., 2011).

Aril development has been divided into stages by some authors, and ontogenetic descriptions suggest that it is a pre-anthesis event originating during megagametogenesis from periclinal divisions of epidermal cells of the funiculus, followed by anticlinal divisions, forming a ring or collar-like structure surrounding the ovule (Kloos and Bouman, 1980; Rodriguez-Riaño et al., 2006). The specific stage of ovule development in which the aril initiates is not very clear in most of the reports. The first divisions might be observed between the tetrad formation stage, when integuments are elongating toward the nucellus, and the beginning of megagametogenesis, when the outer integument has already enveloped the inner integument and the nucellus, forming the micropyle (Raju, 1956; Singh, 1962; Dathan and Singh, 1973; Grear and Dengler, 1976; Kloos and Bouman, 1980; Rodriguez-Riaño et al., 2006).

## Molecular Mechanisms Controlling Integument Initiation and Growth

As mentioned, the aril initiates during ovule development after the emergence and growth of integuments, resembling its development and exhibiting similar patterns of polarity. Thus, to speculate on whether the aril is an extra integument, and which molecular mechanisms might be involved in its identity and development, one should look closely to the molecular basis at the integument initiation and growth.

The development of the ovule in plants has been well characterized in model species, such as *Arabidopsis* and *Petunia*, through molecular genetic studies. Several genes involved in different events of ovule development where identified through mutant screening, as reviewed by Angenent and Colombo (1996), Gasser et al. (1998), and Schneitz (1999). The results obtained from mutant characterization, patterns of gene expression, and transcriptomic analyses in the last two decades allowed for the elucidation of regulatory networks controlling the initiation and development of integuments. Most of the genes characterized encode transcription factors, and molecular studies have been performed to better understand the means by which these factors act, and how they interact regulating integument morphogenesis.

**FIGURE 2:**
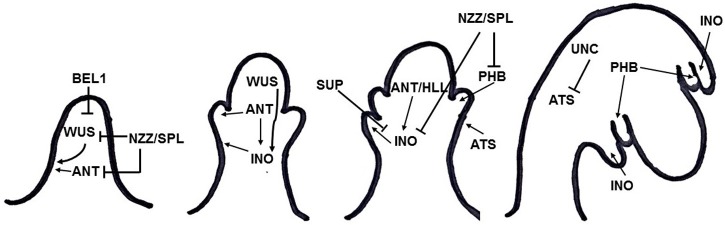
**Summary of gene interactions during early integument development (adapted from Skinner et al., 2004).** Modulation of these interactions might cause the formation of supernumerary integument and/or aril-like structures.

Integument formation marks the transition from the earlier established proximal-distal axis of the ovule primordia to an additional adaxial/abaxial polarity axis. Integument initiation is characterized by epidermal cell proliferation in a region between the nucellus and the funiculus. The putative transcriptional regulator NOZZLE/SPOROCYTELESS (NZZ) is required for maintaining the homeobox gene *WUSCHEL* (*WUS*) expression limited to the nucellus (**Figure 2**) (Sieber et al., 2004). Another factor restraining *WUS* in the nucellus is the interaction of BEL1 (a homeodomain protein) with an integument identity protein complex that represses *WUS* in the chalaza, and activates *INNER NO OUTER* (*INO)* for outer integument development (**Figure 2**) (Brambilla et al., 2008). *WUS*, in turn, is sufficient to induce integument formation from the underlying chalazal tissue, since it generates downstream signals inducing meristematic activity even where it is not expressed (Gross-Hardt et al., 2002). An evidence for this is the induction of ectopic structures resembling integuments at the flanks of the funiculus, when *WUS* is ectopically expressed in the chalaza, under the control of the *AINTEGUMENTA* (*ANT*) promoter (Gross-Hardt et al., 2002). Thus, ectopic WUS expression caused by natural gain-of-function mutation(s) might be involved in the evolutionary origin of supernumerary integuments and, therefore, in structures resembling arils.

Additionally, NZZ is known to restrict both the homeodomain-leucine zipper gene *PHABULOSA* (*PHB*) in the abaxial domain of the chalazal region where the inner integument initiates (Sieber et al., 2004), and INO, which is responsible for outer integument differentiation (**Figure 2**) (Schneitz et al., 1997; Villanueva et al., 1999). *INO* expression, in turn, is restricted to the outer integument by *WUS* and, more specifically, to the abaxial side, where it is repressed by *SUPERMAN* (*SUP*). Thus, INO and SUP are responsible for the asymmetric growth of the outer integument (**Figure 2**) (Meister et al., 2002). *BEL1*, *ANT*, and *HUELLENLOS* (*HLL*) also participate directly or indirectly in *INO* negative spatial regulation (Villanueva et al., 1999). These antagonistic relations control integument polarity.

An additional mutant in which both integuments are present, but exhibits aberrant features is worth mentioning. The *unicorn* (*unc*) mutation results in excrescences emerging from the outer integument (Schneitz et al., 1997). Later on, *UNC* was found to encode an AGC VIII kinase that directly interacts with and represses the activity of ABERRANT TESTA SHAPE (ATS), a transcriptional regulator belonging to the *KANADI* family (**Figure 2**) (Enugutti et al., 2012; Enugutti and Schneitz, 2013). Thus, ectopic expression of *ATS* would provide another mechanism by which additional initiation and growth of integument-derived tissue may occur, therefore indicating an alternative possible molecular mechanism underlying the evolutionary origin of aril or aril-like structures.

Considering the amount of information on regulatory networks for integument initiation and growth, along with the fact that most of these mechanisms are conserved among different taxa, and the known morphoanatomy of arils, it becomes possible to identify the initial cues on the molecular basis of aril origin and development.

## Molecular Aspects of the “Ripening” of Fleshy Seed Structures

The development of fleshy seed structures such as the aril can be divided in three main stages: (1) initiation, which includes cell proliferation; (2) growth, with cell expansion, mainly; and (3) accumulation of storage products, which would be equivalent to a “ripening” stage. As we are assuming a similarity between integument and aril development, we considered the first two stages in the previous section, and we will now consider the third stage.

Since gymnosperms do not form ovaries that will develop into fruits after fertilization, many species developed fruit-like fleshy structures around their seeds to attract frugivorous animals that act as seed dispersers (Herrera, 1989). Because of its importance in the formation of reproductive structures in both gymnosperms and angiosperms, the involvement of MADS-Box genes in the development of fleshy structures was investigated in *Ginkgo biloba* and *Taxus baccata*, both gymnosperms (Lovisetto et al., 2012, 2013, 2015a), and in *Magnolia grandiflora*, a basal angiosperm (Lovisetto et al., 2015b). Gene expression analyses showed that *AGAMOUS*, *AGL6* (a gene phylogenetically close to the *SEPALLATA* clade), and *TM8-like* genes, are involved in the development of fleshy structures in both the sarcotesta of *Ginkgo* and the aril of *Taxus*, regardless of their anatomical origin (Lovisetto et al., 2012). Moreover, activated forms of AGL6 (AGL6::VP16) triggered ectopic outgrowths on the surface of reproductive structures in *Arabidopsis* (Koo et al., 2010). A subfamily of MADS-Box, the *B-sister* genes, is believed to be required for the correct development of ovule and seed, with their expression analyzed in the gymnosperms mentioned above (Lovisetto et al., 2013). The pattern of gene expression differed between these two species, being weaker throughout aril development in *Taxus*, indicating that the involvement of B-sisters in the formation of fleshy fruit-like structures might be dependent of their origin. In *Magnolia*, with a fleshy tissue also originating from the seed tegument, *AGAMOUS*, *AGL6*, *SEPALLATA*, and *B-sister* were also detected during the sarcotesta formation and growth (Lovisetto et al., 2015b).

There is evidence that a common set of genes was recruited independently in distantly related taxa, regulating the development of all fleshy structures, regardless of their anatomic origin in, both, gymnosperms and angiosperms. Accordingly, a group of tomato MADS-box genes have been implicated in fruit ripening, including members of the *SEPALLATA* and B-sister clades (Vrebalov et al., 2002; Yasuhiro, 2016). Altogether, these observations suggest that fleshy tissues that undergo physiological changes that involve tissue softening, pigmentation, and accumulation of sugars, aroma, and flavor (or “ripening syndrome,” in general), appeared independently in fruits and seeds but are likely to be regulated, at the molecular level, by conserved pathways.

## *Passiflora* As A Suggested Model System to Study Aril Development

**FIGURE 3:**
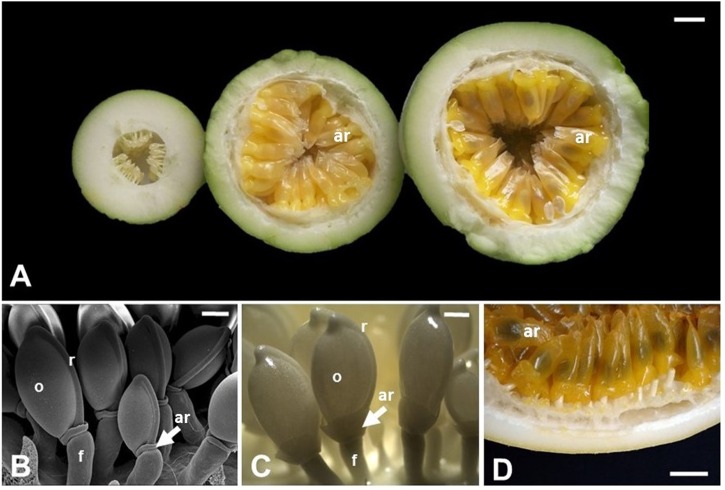
**Aril development in *Passiflora edulis*.** **(A)** Longitudinal sections of fruits at 10, 42, or 49 days after pollination, showing arils at different developmental stages. The arils (yellow/orange) completely cover the seeds, which are observed as darker structures inside the arils. **(B)** Scanning electron microscopy of seeds at 2 days after pollination. **(C)** Seeds at 12 days after pollination. **(D)** Detail of a mature fruit in cross section, where the arils cover the whole seed. ar, aril; f, funiculus; o, ovule; r, raphe. Bars: **A,D** = 1 cm; **B** = 0.5 mm; **C** = 1 mm.

Among the angiosperms, species belonging to *Passiflora* are noteworthy regarding their aril, which are often cited in anatomical and morphological literature as an example of a true aril. *Passiflora* is the largest genus of the family Passifloraceae with over 500 species, mostly originated in neotropical regions, with hundreds of species throughout Latin America (Kugler and King, 2004; Ulmer and MacDougal, 2004). *Passiflora* also include commercial species, such as *P. edulis*, *P. alata*, and *P. incarnata*, which are important for their ornamental, medicinal, and food values, the latter given specifically by the aril in which juice is produced and accumulated. Typically, the fruits of *Passiflora* are indehiscent berries, rarely a dehiscent capsule, very variable in shape, size, and color, and in general produces a mucilaginous or aqueous acidic pulp, forming a cupuliform or saccate aril, covering each of numerous seeds (**Figure 3**) (Cervi, 1997; Dhawan et al., 2004). Passionfruit propagation is mainly carried out by seeds (Pereira and Dias, 2000), and the aril works as a reward for its dispersing agents (Ulmer and MacDougal, 2004), therefore, being directly related to the reproductive success of the wild species (Fenster et al., 2004), which highlights the ecological importance of this structure.

Studies about the aril ontogeny in *Passiflora* are scarce, although it has been addressed in descriptions of the embryology or seed coat structure of Passifloraceae (Raju, 1956; Singh, 1962; Dathan and Singh, 1973; Kloos and Bouman, 1980). The first mention of aril initiation in *Passiflora* is from *P. suberosa* and describes it as a ring around the distal area of the funiculus (Kratzer, 1918 in Kloos and Bouman, 1980). Later studies also refer to the aril primordium as a rim, collar or ring around the funiculus in several species of the family, *P. calcarata* (Raju, 1956; Singh, 1962), *P. foetida* (Singh, 1962), *P. caerulea*, *P. molissima* (Dathan and Singh, 1973), and *P. edulis* (Dathan and Singh, 1973; Corner, 1976). These authors describe the origin of the aril as dermal, epidermal or hypodermal, and are not precise whether it develops from the funiculus, exostome, hilum, micropyle or raphe. A more detailed case study of aril development was performed using *P. suberosa* and *T. ulmifolia*, (Kloos and Bouman, 1980). According to this description, the aril is initiated during megagametogenesis by periclinal and anticlinal divisions of dermal cells, forming a rim around the funiculus from the raphe to the outer integument region at the micropyle (Kloos and Bouman, 1980). Differences among species occur mainly after fertilization. The aril of *P. suberosa* continues to grow, covers the micropyle, and by division of its apical cells, equally envelopes the developing seed, while the aril of *T. ulmifolia* grows unilaterally leaving the exostome exposed.

In spite of these descriptions of the initiation and development of the aril in *Passiflora* species, the molecular mechanisms implicated in these processes have not been described yet. Few studies addressed gene expression in *Passiflora* arils, such as the analysis of differential expression among *PeETR1*, *PeERS1*, and *PeERS*. These genes encode proteins involved in ethylene perception in *Passiflora* fruit tissues, with higher levels of mRNA in arils than in seeds during fruit ripening (Mita et al., 1998; Mita et al., 2002). Nevertheless, these focused mainly on fruit ripening and, therefore, in genes involved in later aril developmental stages, and not in the identity and differentiation of this specialized structure.

Although in recent decades there has been a breakthrough in genome sequencing and genomic data analysis from crop species, efforts for entire genome sequencing were not done in *Passiflora* species, and very little is known about the genomics of this genus. The currently available sequence data in public databases are molecular markers used in phylogenetic and genetic diversity studies, such as microsatellites (Oliveira et al., 2005, 2008; Pádua et al., 2005; Cazé et al., 2012; Cerqueira-Silva et al., 2012, 2014), and internal transcribed spacers (Muschner et al., 2003; Yockteng and Nadot, 2004). On the other hand, specific transcript and genomic libraries for *Passiflora* have been constructed: a database of expressed sequence tags (ESTs) from libraries derived from *P. edulis* and *P. suberosa* reproductive tissues (Cutri and Dornelas, 2012), and a large-insert bacterial artificial chromosome (BAC) library of *P. edulis* (Santos et al., 2014). These are very resourceful for genomic studies allowing a greater understanding of gene structure and function, and the process of differentiation of complex morphological characters, which provide the diversity found among plants, such as the aril. Another useful resource that should aid these functional and developmental studies is the availability of genetic transformation and *in vitro* regeneration protocols for *Passiflora* species. Such protocols where generated by the large number of studies aiming at the genetic improvement of passion fruit, that have been carried out since the 1990s (Cerqueira-Silva et al., 2014), mainly to obtain transgenic plants resistant to the woodiness virus in *P. edulis* (Manders et al., 1994; Alfenas et al., 2005; Trevisan et al., 2006; Monteiro-Hara et al., 2011), and *P. alata* (Correa et al., 2015). Several protocols for *in vitro* regeneration via organogenesis or somatic embryogenesis for a large number of *Passiflora* species where established aiming at germplasm preservation, and recovery of transgenic plants, as reviewed by Vieira and Carneiro (2004) and Otoni et al. (2013). Although designed for breeding purposes, these methodologies are important tools to study the molecular basis of aril development. Novel genomic editing tools, such as the CRISPR/Cas9 technology, will also help in the genetic and molecular analysis of aril development.

## Conclusion

Arils are accessory seed structures present in both gymnosperms and angiosperms, being important for seed dispersal, and might possess economic importance. Nonetheless, aril evolutionary origin and ontogenesis are largely unknown, with, both, structural and molecular information lacking and needed. Here we established parallels between ovule integuments and arils that might help the design of further studies. Our testable statements need a novel model species, since the traditional plant models do not develop arils. We postulate that *Passiflora* species are good candidates for such needed model.

## Author Contributions

SRS, MD, and AM designed the initial manuscript. SRS wrote the initial draft of the manuscript and conceived the figures. SRS, MD, and AM contributed reviewing and discussing the manuscript to produce its final version.

## Conflict of Interest Statement

The authors declare that the research was conducted in the absence of any commercial or financial relationships that could be construed as a potential conflict of interest.
